# Paternalism vs. Autonomy: Are They Alternative Types of Formal Care?

**DOI:** 10.3389/fpsyg.2019.01460

**Published:** 2019-06-28

**Authors:** Rocío Fernández-Ballesteros, Macarena Sánchez-Izquierdo, Ricardo Olmos, Carmen Huici, José Manuel Ribera Casado, Alfonso Cruz Jentoft

**Affiliations:** ^1^Department of Psychobiology and Health, Autonomous University of Madrid, Madrid, Spain; ^2^Department of Psychology, Comillas Pontifical University, Madrid, Spain; ^3^Department of Methodology, Autonomous University of Madrid, Madrid, Spain; ^4^Department of Social Psychology and Organizations, National University of Distance Education, Madrid, Spain; ^5^Faculty of Medicine, Complutense University of Madrid, Madrid, Spain; ^6^Servicio de Geriatría, Hospital Universitario Ramón y Cajal, Instituto Ramón y Cajal de Investigación Sanitaria, Madrid, Spain

**Keywords:** caregiving, paternalism, autonomy, type of care, person centered care

The field of aging shows an extraordinarily high variability, usually classified as pathological, normal, and successful aging (Rowe and Kahn, 1987). Some of these ways of aging require certain amount of care, from successful aging promotion to pathological intensive assistance. Moreover, care of older adults is a broad, complex, and heterogeneous field in which an older person interacts with other persons, mainly family members and/or professionals (that is, caregivers) in a specific context, receiving goods, such as health or social care, welfare, and/or protection support when needed or other less defined types of goods, such as health education, social support or a variety of shared recreational activities. The type of care or social interactions provided by the caregiver depends on the care required by the older adult's physical, psychological or social conditions in interaction with the caregivers' knowledge, abilities of care and views of aging taking place in an institutional or natural environment. In this complex human situation, two main perspectives of care have been called: *paternalist* vs. *person centered or autonomist*, being usually considered *antagonist* ways of care (Brownie and Nancarrow, 2013).

As emphasized by Gallagher (1998), paternalist care is characterized by a dominant *attitude* of superiority, “We know, you don't,” usually is being expressed by caregiver through overprotection over the care recipient.

Conversely, modern social and health care management, from an equalitarian position, includes the patient in the decision making process, under the assumption that the patient *is able* to participate in the decision making process of care (see also Rodriguez-Osorio and Dominguez-Cherit, 2008), not only as new managerial way to considering patient, as a client, but in order to obtain or reinforce client/patient autonomy (Langer and Rodin, 1976; Pavlish et al., 2011; Bercovitz et al., 2019).

It has been emphasized that these two apparently polar orientations can be compatible in the care context (Perry and Applegate, 1985), because they depend on the characteristics of the subject of care: cognitive and physical functional conditions, state of consciousness and understanding, legal situation, etc. Here we will discuss to what extent these two types of care could be and must be compatible depending on certain individual care-recipient characteristics.

## Paternalist Care

The etymology of *paternalism* is based on the Latin word *pater* (“father”) and the patriarchal cultures in which the father is the head of the family, an authority figure responsible for the welfare of family members and other subordinates and dependents. The term *paternalism* appeared in the late 19th century as part of a critique predicated on the inherent value of personal liberty and autonomy. It is associated with attitudes of overprotection that are commonly understood as an infringement of the personal freedom and autonomy of a person (or class of persons) with a beneficent or protective intent. In the field of health and social care, paternalism includes the confrontation between individual personal needs and human rights on one hand and social overprotection and care on the other (Thompson, 2017).

Szerletics (2015) argues that paternalism can be defined by its motive, which implies benevolence, “benevolent decision-making in another's best interests” (Tuckett, 2006), therefore, from this point of view, interventions that promote “the good or welfare of the agent who is coerced” (Husak, 1981) can be justifiable, no matter how harsh they interfere with personal autonomy. When formal caregivers underestimate an old person's capabilities, do not treat him/her as an adult, provide unnecessary help and attempt to restrict his/her activities, caregivers overprotect the care recipient who does not ask for nor requires protection (Thompson and Sobolew-Shubin, 1993a,b; Thompson et al., 2002; Cimarolli et al., 2013; Ugarhood et al., 2017) this would be a true expression of paternalism. Nevertheless, depending on the characteristics of the subject of care, he or she may require protection or even overprotection or no protection at all. Therefore, a paternalist type of care implies that the individual is not considered as an autonomous person who is requiring protection or overprotection because his/her age must be defined properly considering needs in the recipient and not caregivers (mis)perceptions or interpretations. The most important threat of paternalistic attitudes and overprotection behaviors are their likely consequences: the older adult's reduction of autonomy/capabilities (e.g., Lawton, 1989; Thompson and Sobolew-Shubin, 1993a,b; Thompson et al., 2002; Cimarolli et al., 2013), therefore, acting as a self-fulfilled prophecy (Little, 1988; Hummert et al., 1995; Antonucci, 1996).

Also, we can find studies focused on overprotection and its negative effects in the family, showing a perverse effect on children's mental health (Anderson and Coyne, 1991; Bögels and Brechman-Toussaint, 2006; Sanders, 2006; Hemm et al., 2018).

## Person Centered or Autonomy Care

The emphasis on autonomy in the field of care, as Whal et al. (2012) have pointed out, started from an interactive model of care based on the client's competence. Thus, in the person/environment interactional theory posited by Lawton and Nahemow (1973), two interacting factors seem to be mediating the type of care in older adult contexts: the level of the older adult's competence, frailty, dependency and/or cognitive impairment are mediated by environmental pressures as well as by the social group holding negative stereotypes and ageist attitudes and behaviors (Lawton and Nahemow, 1973). Within this complex situation, it is important to respect the person's degree of autonomy. Autonomy, from its Greek origins, means self-rule or self-governance (*auto* = self, *nomos* = rule or governance), that is, the person's self-determination of, and self-governance over, his/her actions, as well as the ability to formulate and carry out a life plan.

In recent decades we find approaches considered alternatives to the traditional paternalist model. The Person-Centered Care, arising from Carl Rogers' theory about human growth (Rogers, 1959), which is based on the assumption that older person functioning is not the product of age and/or illness but the results of the interaction between the characteristics of individuals and their psycho-social environment, based on strong empirical support (Brownie and Nancarrow, 2013; Barbosa et al., 2015; Fernandez Ballesteros et al., 2016).

Similarly, The Patient Activation Theory (Hibbard and Mahoney, 2010), based on the concepts of self-efficacy (Bandura, 1978, 1994), locus of control (Rotter and Mulry, 1965; Rotter, 1966) and in the transtheoretical model of change (Prochaska and Velicer, 1997) focuses on “patient engagement” (Graffigna et al., 2017a), the potential of the persons when becoming protagonists of their care management, promoting their knowledge, skill, and confidence (Graffigna et al., 2017a,b).

Taking into consideration these two perspectives, paternalism and autonomy could both be present to some extent in care contexts, and both could be implicitly or explicitly shown by attitudes and behaviors exerted by family members, professional caregivers (physicians, nurses, social workers, psychologists, voluntary caregivers, etc.), or even general stakeholders. But, to what extent these two types of formal care are independent or can be related to other conditions, such as the degree of the older adult's cognitive and physical functionality?

## Two Types of Care in Two Types of Context

In an attempt to better understand the prevalence and appraisal of these two types of care among professionals in different settings with different types of clients'needs, we developed The Paternalist/Autonomist Care Assessment (PACA) (Fernández-Ballesteros et al., submitted) composed by two subscales: “PACA-Appraisal” reflects to what extent its 30 items are describing forms of treating older adults, and “PACA-Occurrence” refers to what extent a given form occurs in a given center. In the development process, through exploratory and confirmatory factorial analysis of both measures, as expected, two factors were identified, that we named *Overprotection* and *Autonomy*.

Some of the *Overprotection* items included were: “Even if the older person is against it, the caregiver should do what he thinks is best for their health,” “When necessary, older people should be urged to follow the treatment proposed by the doctor and if they resist, it should be done without them realizing it,” “Everything that older person has problems with should be done for them.” While the factor *Autonomy* included items like the following: “Older people should have the opportunity to choose the activities to do each day,” “The older person must be the one who decides whether or not to undergo surgery,” “If the daily routine of an older person needed changing, the reasons why would have to be carefully explained to them.”

In order to test to what extent the two types of care appear in several contexts, the PACA was administer to formal caregivers (*N* = 160) working in Day Care Centers for older persons (*N* = 70), where physical and cognitive rehabilitation is provided, and to caregivers working in Senior Citizen Centers (*N* = 90), where only learning and leisure activities are organized. This study was approved by the Ethics Committee of Autonoma University of Madrid (November 2014). All subjects gave written informed consent in accordance with the Declaration of Helsinki.

**Figure 1:**
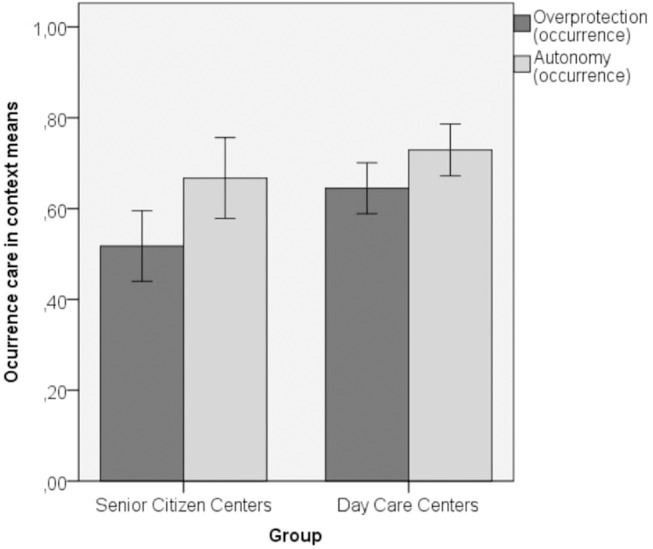
Means and SD of overprotection and autonomy in the occurrence measures.

Trying to learn more about the sources of variability of this study—Factors (Overprotection and Autonomy) and Centers assessed (Day Care Center and Senior Citizen Center) a split-plot ANOVA has been conducted for the Occurrence measure (that is, the observed behavior in the context). The within factor was Overprotection and Autonomy and the between factor was the center (Day Health and Senior Citizen centers). The results were quite different (Figure 1). Although the interaction effect was not significant [*F*_(1, 116)_ = 1.101, *p* = 0.295, η^2^ = 0.009], simple effects showed that in Senior Care Centers, the Autonomy mean was significant higher than Overprotection mean [*F*_(1, 116)_ = 11.367, *p* = 0.001], but in Day Health Care Centers no significant differences were found between Autonomy and Overprotection means [*F*_(1, 116)_ = 3.723, *p* = 0.056]. Moreover, the Overprotection mean was significant higher in Day Health Care Centers than in Senior Care Centers (*p* = 0.009), but the Autonomy factor did not differ significantly between the two centers (*p* = 0.240). This is an empirical evidence that the observed occurrence measure of Overprotection and Autonomy yields a significant difference that only occurs in Senior Citizen Centers, but not in Day Health Care Centers, where there were no differences in the two factors (e.g., Overprotection and Autonomy do not differs). Thus, higher functioning persons attending Senior Citizen centers seem to elicit higher Autonomy while no differences were found for lower functioning persons in need of Day Care.

In conclusion, our results yielded by the PACA suggest that *paternalist* and *autonomist* care factors can operate independently from each other and those formal caregivers may be fitting their care behaviors depending on older adults' level of functioning in a formal care context. In fact, in Day Care, where there is a high variability in users functional status, both types of care (Paternalist and Autonomist) exist in approximately the same proportion, but in Senior Citizen Centers, with a high homogeneity of high functioning users since, the Autonomist style model of care predominates over a Paternalist care.

In sum, we may assume that *paternalist* and *autonomist* care factors can operate independently from each other and that formal caregivers may be fitting their care to older adults functioning in the Care context. As already pointed out, aging has a wide variability requiring various level of protection as well as autonomy promotion and, similarly as in families with children with different physical, mental and emotional resources, in care contexts older clients have several needs depending of their resources (Anderson and Coyne, 1991; Thomasgard and Metz, 1993; Kim et al., 2003).

Although paternalistic attitudes have been considered intrinsically wrong, protection (but never overprotection that is providing care without considering the receiver's needs) may depend on the functionality of the older adults been cared for. Also, although the promotion of autonomy is intrinsically right, it may be adjusted to the individual baseline characteristics, taking into consideration that a very high level of autonomy demand could overcome the individual base line, producing anxiety, and suffering. Therefore, more research is needed to provide evidence regarding which mode of care is more beneficial and fitting in each context and our PACA instruments have been developed with this purpose.

## Author Contributions

RF-B: study concept and design, led the study, writing, and approval of manuscript. MS-I: data collection, preparation, and revision of manuscript. RO: preparation of manuscript. CH: expertise on stereotypes and critical review of manuscript. JR: critical review of manuscript. AC: preparation of manuscript. All authors: revision of manuscript.

### Conflict of Interest Statement

The authors declare that the research was conducted in the absence of any commercial or financial relationships that could be construed as a potential conflict of interest.
